# Strain mapping using compressed sensing accelerated 4D flow MRI—Potential for detecting coactivation in thigh muscles

**DOI:** 10.3389/fphys.2025.1583024

**Published:** 2025-05-23

**Authors:** Usha Sinha, David Maldonado, Vadim Malis, Ning Jin, Edward Smitaman, Ramon Sanchez, Christine Chung, Shantanu Sinha

**Affiliations:** ^1^ Physics, San Diego State University, San Diego, CA, United States; ^2^ Department of Radiology, University of California San Diego, San Diego, CA, United States; ^3^ Cardiovascular MR R&D, Siemens Medical Solutions USA, Inc, Cleveland, OH, United States; ^4^ Division of Musculoskeletal Imaging, Department of Radiology, University of California San Diego, San Diego, CA, United States; ^5^ Muscle Imaging and Modeling Lab, Department of Radiology, University of California San Diego, San Diego, CA, United States

**Keywords:** thigh muscle strain mapping, compressed sensing accelerated 4D flow MRI, coactivation of hamstrings, diffusion tensor imaging (DTI), contractile strain

## Abstract

**Introduction:**

Deterioration in knee extensor function is significantly related to decline in functional mobility, stability and proprioception increasing risk of injury and falls. Coactivation of the hamstrings has been reported in normal and neuromuscular conditions during knee extension emphasizing coordinated muscle activation through range of motion. This study evaluated a prototype compressed sensing accelerated 4D Flow (CS-4DFlow) sequence for volumetric MRI strain mapping of the entire thigh during isometric contraction.

**Methods:**

Dynamic imaging (at 30% and 45% Maximum Voluntary Contraction (MVC)) was performed with the CS-4DFlow sequence integrating a L1-regularized wavelet-based compressed sensing reconstruction. Strain tensors were computed from displacements tracked from the velocity data. % MVC and muscle related differences (within hamstring and quadriceps and between muscle groups) in normal, shear and volumetric strain were statistically analyzed.

**Results:**

Transverse asymmetry of deformation was seen in all the thigh muscles during isometric contraction. Significant differences in the strain indices with %MVC was seen in the quadriceps but not in the hamstrings. The averaged values of the quadriceps and hamstring muscles showed significant differences between the two muscle groups and with %MVC in all the strain indices. Hamstring strain was around 50% of the quadriceps strain signifying a high level of coactivation. Coactivation was also visually confirmed by comparing the directions of the contractile strain and fibers in the two muscle groups.

**Discussion:**

The current study establishes the feasibility of volumetric strain imaging of the thigh under isometric contraction. The ability to map all muscles allows evaluation of coactivation of the hamstrings with potential for application to conditions such as osteoarthritis and ACL deficiency.

## 1 Introduction

The quadriceps are the extensor muscles of the knee joint and weakness in this muscle group is associated with knee osteoarthritis (Briani et al., 2018). The importance of the extensors is underlined by the fact that their deterioration is significantly related to the decrease in the quality of life of the subject (Brown et al., 1995). Isometric training has emerged as a popular intervention technique to counter quadriceps muscle weakness (Fitzgerald et al., 2003). The quadriceps muscle includes the four heads of the muscle bellies of the vastus lateralis (VL), vastus medialis (VM), vastus intermedius (VI), and rectus femoris (RF). The main knee flexor muscles are the hamstring muscles: the semitendinosus (ST), semimembranosus (SM), and long and short heads of the biceps femoris (LBF and SBF). These muscle groups work in coordination to optimize stability and balance.

Muscle function is most conveniently monitored using Electromyography (EMG) that measures the electrical activity in response to a nerve’s stimulation of the muscle and this technique has been applied to detect neuromuscular abnormalities (Kroczka et al., 2009). Most of these studies are based on surface EMG measurements where the signals are picked up from shallow muscles and from large surface areas that can give rise to cross-talk and suffer from lack of information on the spatial distribution of the electrical activity. Intramuscular EMG provides information on activity from deep muscles as well as from a small area close to the inserted needle but is not routinely used due to its invasive nature (Péter et al., 2019). To date, electromyography is used as a pseudo-outcome for the assessment of muscle performance by measuring the sum of active motor units in the vicinity of the electrodes (Péter et al., 2019).

Ultrasound (US) studies using tissue velocity imaging or speckle tracking have demonstrated the utility of strain and strain rate imaging in characterizing normal and diseased skeletal muscles (Frich et al., 2019; Peolsson et al., 2008). However, US imaging is operator dependent, primarily available only in 2D mode, and speckle tracking requires complex post processing and suffers from low signal-to-noise ratio (SNR). In addition, US imaging is constrained in the field of view and is limited to a few muscle groups as all the muscles cannot be visualized simultaneously. For example, it would not be possible to study both the quadriceps and hamstring muscles during a dynamic contraction of the thigh.

Velocity encoded phase contrast (VE-PC) imaging has been well established as a viable technique for imaging skeletal muscle dynamics (Sinha et al., 2015; Malis et al., 2018; Sinha et al., 2020; Deligianni et al., 2017; Deligianni et al., 2020). The single slice dynamic imaging using 2D VE-PC has been used to study the calf muscle under different contraction paradigms and applied to studying age related differences as well as unloading (Sinha et al., 2015; Malis et al., 2018; Sinha et al., 2020). It has also been applied to thigh muscle imaging to establish feasibility (Oda et al., 2021) and to explore age related differences in muscle strain during electrical stimulation (Deligianni et al., 2020). Most of the earlier studies have been performed using a 2D technique that limited the acquisition to a single slice during the dynamic contraction. The medial gastrocnemius (MG) has been the focus of the dynamic studies since this muscle has a fairly simple fiber architecture and it is possible to select an imaging plane that contains the muscle fibers in the plane of the image (Sinha et al., 2015; Malis et al., 2018; Sinha et al., 2020). This allowed the study of the true 3 × 3 strain tensor as a 2 × 2 strain tensor. However, this approach does not lend itself to other muscles such as the soleus or thigh muscles that have a more complex fiber architecture. In an attempt to reduce scan times, compressed sensing was applied to a 2D VE-PC sequence that reduced scan times by a factor of 4 (Malis et al., 2020a). The reduced scan time enabled sequential multi-slice acquisition and at multiple % MVC. The latter sequence was then applied to studying age related effects of muscle force loss and muscle strain in the plantar flexors at three %MVCs. Lower strains/strain rate and shear strains/strain rate were seen in the soleus and in the MG in the senior cohort (Malis et al., 2020b). While the 2D compressed sensing sequence allowed scan times to be reduced significantly enabling sequential multi-slice acquisition, it still lacks the flexibility and the SNR advantage of a true 3D acquisition.

Mazzoli et al. used a CS-4DFlow sequence (acceleration factors up to 6.41) to extract the 3 × 3 strain rate tensor in the calf muscles and showed that the accelerated sequences yielded strain rate values that were not significantly different from the reference unaccelerated image (Mazzoli et al., 2018). A prototype 4D (3-dimensional time resolved and with 3 directional velocity encoding) flow sequence integrating compressed sensing with an undersampling factor of 7.7, CS-4DFlow, has been implemented recently to study flow in blood vessels (velocities of the order of 100 cm/s) (Ma et al., 2019). There is one earlier study based on the CS-4DFlow sequence that used neuromuscular electrical stimulation to map the strain in soleus and MG muscles (Santini et al., 2019). 3D strain measurements on calf muscle from sequential 2D dynamic acquisitions have shown larger changes in soleus (compared to the MG) going from 0% to 30% Maximum Voluntary Contraction (MVC) while the MG showed the larger increase in the 30%–60% MVC range (Malis et al., 2020b). These MRI based findings are in line with EMG studies on the relative activation of slow twitch soleus at low loads that switches to the predominantly fast twitch MG at higher loads (Moritani et al., 1991). While both the quadriceps and hamstrings contain some slow-twitch muscle fibers (Type I), hamstrings generally have a higher proportion of fast-twitch fibers (Type II) compared to the quadriceps. Further, even within the quadriceps, the vastus lateralis has a higher proportion of fast twitch fibers. A dynamic thigh muscle study at different %MVC similar to the dynamic calf muscle strain mapping study would enable load dependent strains to be assessed in different muscles and muscle groups with varying fiber types.

Muscle coactivation or co-contraction is the simultaneous activation of agonist and antagonist muscles. The agonist produces force and/or moment of force in a direction to accomplish a specific task, while the antagonist opposes this action (Latash, 2018). As a result, one of the direct mechanical effects of coactivation within an agonist-antagonist pair is reduction in the resultant forces and moments as compared with those that could be expected in the absence of coactivation. Surface EMG (sEMG) has been extensively used to study coactivation in different agonist-antagonist muscles in a variety of contraction paradigms (Latash, 2018). A prior study found good agreement between EMG data and displacements computed from 2D VE-PC MRI and concluded that the MRI technique maybe used as a noninvasive, high-resolution approach for quantifying and modeling muscle activity over the entire 3-D volume of muscle groups (Csapo et al., 2015). The CS-4D Flow sequence allows coverage of both the quadriceps and hamstring muscles enabling strain mapping to follow muscle function in agonists and antagonists.

In this study, we explore the feasibility of the CS-4DFlow sequence to image the much smaller motion seen in muscle tissue (<1 cm/s). The specific aims are 1) to optimize the CS-4DFlow sequence for dynamic thigh muscle imaging, 2) to acquire dynamic data for the entire thigh volume during isometric knee extension at two %MVC to study load dependent strain differences in the quadriceps and hamstring muscles, 3) to extract 3 × 3 strain tensors and quantify the principal eigenvalues, maximum shear strain and volumetric strain in representative ROIs of the agonist quadriceps muscles and in the antagonist hamstring muscles (co-activation), 4) visualize the direction of the negative strain in the agonist and antagonist muscles at peak force and compare it to the muscle fiber direction; the latter derived from diffusion tensor images acquired geometrically matched to the volumetric strain images, and 5) to explore differences within muscles of the quadriceps and within muscles of the hamstrings, with %MVC, and between the quadriceps and the hamstring muscles (average over all the individual muscles of each group). The ultimate goal is to apply the normative strain and co-activation data to common clinical problems exploring the role of dynamic stabilization and coordinated muscle activation in Anterior Cruciate Ligament (ACL) deficiency (Yanagawa et al., 2002), osteoarthritis (Murphy et al., 2022), and muscle injury characterization and rehabilitation (Silder et al., 2010). It should be noted that recent advances in deep learning methods applied to the areas of image classification (Iqbal et al., 2021), region detection (Iqbal et al., 2020), and reconstruction (Vishnevsky et al., 2020) can be extended to the 4D flow MRI method.

## 2 Materials and methods

### 2.1 Subject and dynamic imaging

Twelve young subjects (24.7 ± 2.3 years old, 10 male, 67 ± 10 kg) were included in this study after written informed consent had been obtained. The criteria for inclusion were that subjects should be active (self-reported as either moderately or more than moderately active) and should have had no surgical procedures performed on the leg. The study was carried out under the approval of the Institutional Review Board of San Diego State University, San Diego, and the studies were conducted in accordance with the local legislation and institutional requirements. The participants provided their written informed consent to participate in this study.

**FIGURE 1:**
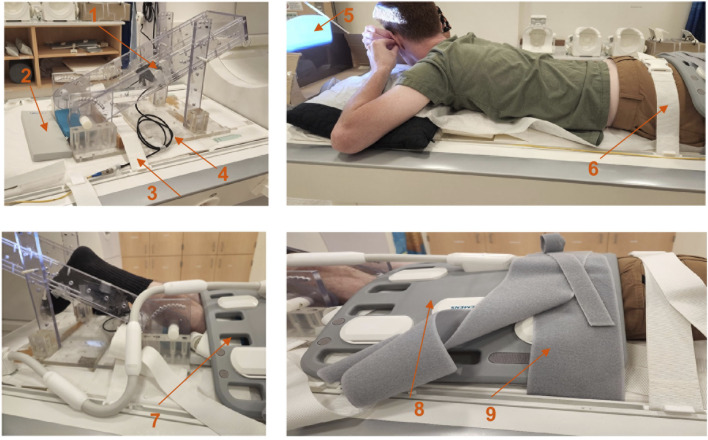
Top left: Details of the force sensor holder; force sensor device is located at the back of the black carbon fiber plate (arrow labeled 1), gray sponge for knee placement (arrow labeled 2), restraint to prevent movement of the foot pedal device (arrow labeled 3), fiber optic cable (arrow labeled 4), projection screen (arrow labeled 5), gluteus muscle restraint (arrow labeled 6), restraint at the knee (arrow labeled 7), body surface coil (arrow labeled 8) and restraint over the body coil (arrow labeled 9).

Magnetic resonance imaging was performed on a 3 T clinical MR scanner (MAGNETOM Prisma, Siemens Healthineers, Forchheim, Germany). The dominant leg for all subjects was chosen for the dynamic scan (ten right dominant, two left dominant). Dominant leg was self-reported as the leg used for kicking a ball. Subjects were positioned feet first in the scanner in the prone position with the anterior part of the thigh horizontal on the spine coil with the body coil wrapped around the posterior part of the thigh. To ensure that the thigh did not move upward during the downward exertion of the tibia (Figure 1), the thigh was stabilized by restrainers over the gluteal muscles and at the knee with a Velcro strap over the surface coil (Figure 1). The tibia rested at a pre-determined angle (∼35° knee flexion) on a firmly anchored carbon fiber plate, which had an optical force transducer on the reverse side (Oda et al., 2021). During isometric contraction the force transducer detected the pressure exerted by the tibia against the transducer plate and the output was coupled to a spectrometer (Fiberscan, Luna Innovations, Roanoke, VA) via a fiber optic cable. The optical signal was converted to voltage and used to trigger the MR image acquisition using custom built software developed in LabView (version 14.0.1.4008. National Instruments Inc, Austin, TX). Prior to the start of the dynamic MRI scans, three trials of the subjects exerting maximum voluntary contractions (MVC) were recorded. Muscle force measured during maximum voluntary contraction had a value of 213.7 ± 78.6 N, averaged over the 12 subjects. Dynamic volumetric images were acquired during two submaximal, isometric contractions at 30% and 45% MVC. Consistency of the contractions was ensured by providing the subject with real-time visual feedback of the actual force generated by the subject superimposed on the target force curve (Figure 1).

### 2.2 Magnetic resonance imaging

The MRI session included a localizer scan followed by a static anatomical scan that spanned the dominant thigh in sagittal slices. The first set of dynamic images were acquired during isometric contraction using the CS-4DFlow sequence at 45%MVC at the same locations as the anatomical scan. This was followed by a static diffusion tensor imaging (DTI) sequence in the sagittal view acquired at the same locations and with the same geometric parameters as the anatomic and dynamic scan. The latter scan was completed in 6 min and this allowed the subject to relax before the second dynamic scan during isometric contraction at 30% MVC.

#### 2.2.1 Compressed sensing accelerated 4D flow imaging

The dynamic imaging was performed with a prototype highly accelerated 4D flow imaging sequence that provided 3-directional velocity encoding for a 3D volume (Ma et al., 2019). Acceleration by a factor of 7.7 was achieved using a L1-regularized wavelet-based compressed sensing reconstruction. The undersampling factor of 7.7 is defined with respect to a fully sampled acquisition along the slice and phase encode directions. The scan was acquired by prospective triggering and the sequence parameters were: TE/TR/FA:: 4.4 m/6.87 m/8^o^/, VENC: 15 cm/s (3 directional encoding), 3 k-space lines per segment, 32 temporal phases, ∼28 sagittal slices (adjusted to span the thigh), FOV: 150(PE) x 300(RO) mm, Matrix size: 80 (PE) x 160 (RO), 5 mm slice thickness with an acquisition time of ∼4.30 min. The period of the dynamic contraction cycle was set to 3 s.

#### 2.2.2 Diffusion tensor imaging

Diffusion tensor images was acquired with the subject at rest. The parameters of the spin echo planar DTI sequence were as follows: TE/TR: 51 m/3300 m, 30 diffusion gradient directions, diffusion weighting b factor of 400 s/mm^2^, FOV: 150(PE) x 300(RO) mm, 5 mm slice thickness, parallel imaging factor: 2, ∼28 sagittal slices. The geometric parameters and anatomic locations of the diffusion data were matched to the dynamic acquisition.

#### 2.2.3 Image analysis of CS 4D flow images

Phase images were corrected for phase shading artifacts and denoised with a 3D anisotropic diffusion filter to yield the velocity images at the three acquired slice locations. Parameters of the anisotropic diffusion filter were: number of iterations *N* = 10, step size δ=3/44 , parameter κ controlling sensitivity to edge gradient strength = 4 and the conductance function *c*_2_ favoring wide regions over small ones, given by Equation 1:

c2∇I=11+∇Iκ2 (1)

where ∇I is the gradient of the input image I. Voxels in the entire volume were tracked to obtain displacements and locations in subsequent temporal frames. The 3D strain images were obtained by taking spatial gradient of the displacements (Equation 2),

$$F=\left[\begin{array}{ccc} \frac{\partial \Delta_{x}}{\partial x} & \frac{\partial \Delta_{y}}{\partial x} & \frac{\partial \Delta_{z}}{\partial x}\\ \frac{\partial \Delta_{x}}{\partial y} & \frac{\partial \Delta_{y}}{\partial y} & \frac{\partial \Delta_{z}}{\partial y}\\ \frac{\partial \Delta_{x}}{\partial z} & \frac{\partial \Delta_{y}}{\partial z} & \frac{\partial \Delta_{z}}{\partial z} \end{array}\right]$$ (2)

the F matrix was then symmetrized and diagonalized as shown in Equation 3 to obtain the Lagrangian (**L**) strain tensor.

$$L=0.5\left(F+F^{T}\right)=\left[\begin{array}{ccc} L_{\lambda_{1}} & 0 & 0\\ 0 & L_{\lambda_{2}} & 0\\ 0 & 0 & L_{\lambda_{3}} \end{array}\right]$$ (3)

The eigenvalues are sorted by ascending values: the lowest value (negative) represents compression (denoted by subscript λ1) and the eigenvector associated with this eigenvalue is approximately along the fiber direction at the peak of the contraction. The highest value (positive) represents the lengthening (denoted by subscript λ3) deformation in the fiber cross-section and the eigenvector associated with this eigenvalue is approximately perpendicular to the fiber direction at the peak of the contraction. The intermediate eigenvalue, L_λ2_ is usually very small, is oriented in the fiber cross-section orthogonal to the directions of the other two eigenvectors. Two invariants (maximum shear and volumetric strain) were calculated from the strain tensor as given by Equations 4, 5.

$$L_{\max}=\frac{2}{3}\sqrt{{\left(L_{xx}-L_{yy}\right)}^{2}+{\left(L_{xx}-L_{zz}\right)}^{2}+{\left(L_{yy}-L_{zz}\right)}^{2}+6\left(L_{xy}^{2}+L_{xz}^{2}+L_{yz}^{2}\right)}$$ (4)

$${\;L}_{\text{vol}}=\frac{\delta V}{V}=L_{\text{xx}}+L_{\text{yy}}+L_{\text{zz}}$$ (5)

The L subscripts in the above two equations refer to the tensor elements prior to diagonalization. The volumetric invariant is represented by *vol* subscript, while the maximum shear strain is represented by the *max* subscript.

In addition, the colormap of the eigenvector corresponding to the negative strain eigenvalue was generated using the convention traditionally used for diffusion tensor colormaps: red hue proportional to the x-component of the eigenvector (Medial- > Lateral), green hue proportional to the y-component of the eigenvector (Anterior- > Posterior), and blue hue proportional to the z-component of the eigenvector (Superior- > Inferior).

The strain values were extracted at the frame corresponding to the max force. ROIs (Supplementary Figures S1A, B) were manually placed in the muscles of interest in the magnitude images of the initial frame and had a size of ∼9.38 mm (w) × ∼ 28.13 mm (h) as shown in Supplementary Figure S1; the width and height were adjusted around this nominal value according to the muscle size. This manually positioned ROI was duplicated in two adjacent slices on either sides of the selected slice to form a volume of interest (VOI). Analysis was performed on the values averaged in these VOIs positioned in the quadriceps (RF, VM, VL and VI)) and hamstring (SBF, LBF, ST, and SM)) thigh muscles.

#### 2.2.4 Image analysis of diffusion tensor images

The DTI data was processed in the following steps using DSI Studio (https://dsi-studio.labsolver.org/). Pre-processing included eddy current correction, smoothing of the baseline and diffusion weighted images, and thresholding to exclude background voxels. The diffusion weighted data was fit to the tensor model using the weighted least squares algorithm and subsequently diagonalized to yield the diffusion eigenvalues and eigenvectors. The largest eigenvalue represents diffusion along the muscle fiber and the corresponding eigenvector represents the muscle fiber direction. The lead eigenvector images representing fiber direction were exported and processed using an in-house program to generate colormaps using the scheme outlined above for strain eigenvector colormaps.

### 2.3 Statistical analysis

The outcome variables of the analysis are the eigenvalues of the tensor as well as the invariants (*L*_*λ1,*_ *L*_*λ2*_ *, L*_*λ3,*_ *L*_*max,*_ *L*_*vol*_). Differences in strain among four quadriceps muscles and changes in strain with %MVC (30% and 45% MVC) were statistically analyzed for all the strain indices. Similar statistical analysis was performed on the four hamstring muscles. The four muscles each of the quadriceps and hamstrings were averaged and differences in strain between these two muscle groups as well as changes with %MVC were statistically analyzed. Normality of data was tested using the Shapiro-Wilk test. Data was normal for the quadriceps *L*_*λ1*_ and for all the strain indices of the averaged quadriceps and hamstring muscle data; a repeated measures ANOVA was used for these variables. All the other indices were non- normally distributed (Shapiro-Wilk test, P < 0.05). Differences between muscles and %MVC for these parameters were assessed for significance using the Friedman test. In the case of significant difference for ‘muscle’, post-hoc analysis was conducted using Bonferroni-adjusted Wilcoxon Signed-Rank Test. Normally distributed variables are reported as mean ± SD and non-normally distributed variables as median ± IQR. For all tests, the level of significance was set at p = 0.05. The statistical analyses were carried out using SPSS for Mac OSX (SPSS 21.0, SPSS Inc., Chicago, IL, USA).

## 3 Results

The dynamic acquisition was sensitive to the restraints placed on the subject, especially the restraint at the knee. Supplementary Video S1 shows the magnitude image of the dynamic sequence and the x-displacement maps for two subjects, one with incorrect (left) and another with effective restraint (right) at the knee respectively. A camera in the magnet bore allowed one to monitor the motion close to the knee; if large motions were visualized, the subject was repositioned with more effective restraints. Other sources of noise in the image arose if the subject was not able to follow the template force curve. Supplementary Figures S2 shows the relative force as a function of the dynamic cycle for all the contractions. Plots are shown for a subject who closely followed the template (left plot) and another who did not comply well with the template curve (right plot). Supplementary Figures S3 shows velocity images without (as acquired) and with denoising while Supplementary Figures S4 shows corresponding strain images computed from the noisy and denoised velocity images. These images show that denoising of the velocity images significantly decreases the noise in the velocity and computed strain images.

**FIGURE 2:**
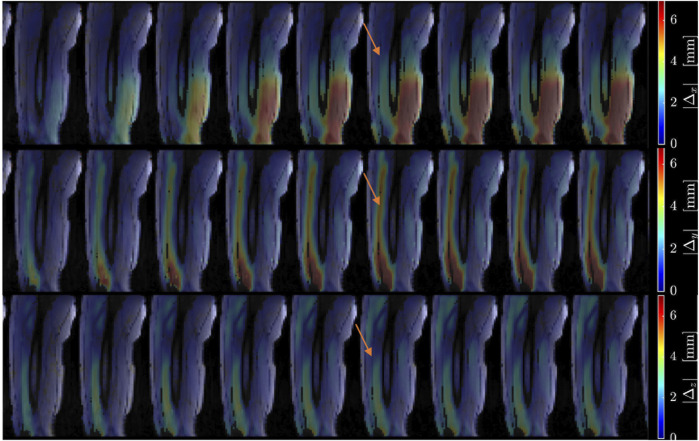
Colormaps of the displacement along the *x*-axis (top panel), *y*-axis (middle panel) and *z*-axis (bottom panel). One slice from the volume of 32 slices is shown here and the temporal frames span (from a total of 32) from 15 to 23 with the peak of the strain around frame 20 (red arrow) for isometric contraction at 30% MVC. The colormaps are superimposed on the magnitude images at each temporal frame; the color-legend is shown on the right.

**FIGURE 3:**
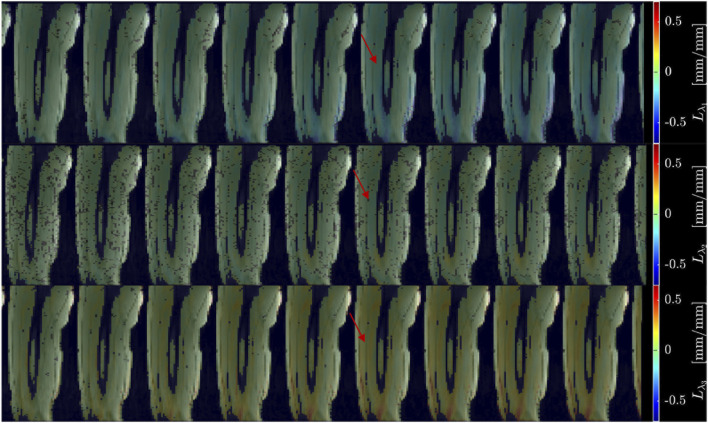
Colormaps of the strain eigenvalues: *L*_*λ1*_ (top panel), *L*_*λ2*_ (middle panel), *L*_*λ3*_ (bottom panel). *L*_*λ1*_ is the negative eigenvalue image while *L*_*λ3*_ is the positive eigenvalue image. The second eigenvalue, *L*_*λ2,*_ is much smaller than the other two. One slice from the volume of 32 slices is shown here and the temporal frames span (from a total of 32) from 15 to 23 with the peak of the strain around frame 20 (red arrow) for isometric contraction at 30% MVC. The colormaps are superposed on the magnitude images at each temporal frame, the color-legend is shown on the right (negative: blue hues, positive: red hues).

**FIGURE 4:**
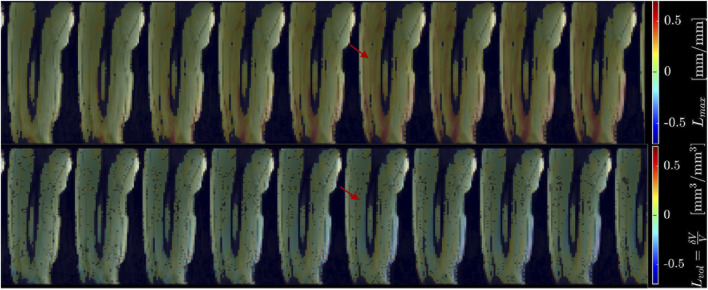
Colormaps of the strain invariants: *L*_*max*_ (top panel), *L*_*vol*_ (bottom panel). *L*_*max*_ is the maximum shear strain while *L*_*vol*_ is the volumetric strain. The values of *L*_*max*_ are higher than the absolute values of the normal strains. *L*_*vol*_ is small as would be expected for a nearly incompressible tissue like muscle. One slice from the volume of 32 slices is shown here and the temporal frames span (from a total of 32) from 15 to 23 with the peak of the strain around frame 20 (red arrow) for isometric contraction at 30% MVC. The colormaps are superposed on the magnitude images at each temporal frame, the color-legend is shown on the right (negative: blue hues, positive: red hues).

Figure 2 shows displacement maps (Δx, Δy, and Δz) of one slice (out of 24 slices in the volume) through 9 temporal frames (15-23 frames out of 32) of the isometric contraction cycle for one subject. The peak of the displacements occurs around frame 20, which is close to the peak of the force curve. The larger displacements in the x direction may arise from gross motion close to the knee region since the downward force exerted by the tibia causes the knee to rise. The large values of y-displacement along the aponeurosis between the RF and VI muscles are clearly evident near the peak of the force curve. Strain maps for the same subject are shown in Figure 3. The panels from top to bottom are the strain colormaps of L_λ1,_ L_λ2_ and L_λ3_ respectively for the same anatomical location and temporal frames as shown in the displacement maps. The larger values of the negative and positive strains in the quadriceps muscles (compared to hamstring muscles) and along the aponeuroses as well as the small strain values in the L_λ2_ colormap can be readily visualized. The colormaps of the invariants *L*_*max*_ and *L*_*vol*_ are shown in Figure 4 for the same anatomical slice and temporal frames as shown in Figures 2, 3. Visually, the maximum shear strain maps have the largest absolute values of the strain (compared to *L*_*λ1,*_ and *L*_*λ3*_ colormaps) with larger values along the aponeuroses as in the *L*_*λ1*_ and *L*_*λ3*_ maps. The *L*_*vol*_ colormap shows low values as would be expected for an incompressible tissue like muscle since it is the relative change in volume. Supplementary Video S2 shows the strain maps through the dynamic cycle (*L*_*λ1*,_ *L*_*λ2*_, *L*_*λ3,*_ and *L*_*max*_). Supplementary Video S3 shows the compressive strain through the dynamic cycle for select slices in the thigh volume. The spatial pattern of the compressive strain in the different thigh muscles can be seen as the volume is panned.

**FIGURE 5:**
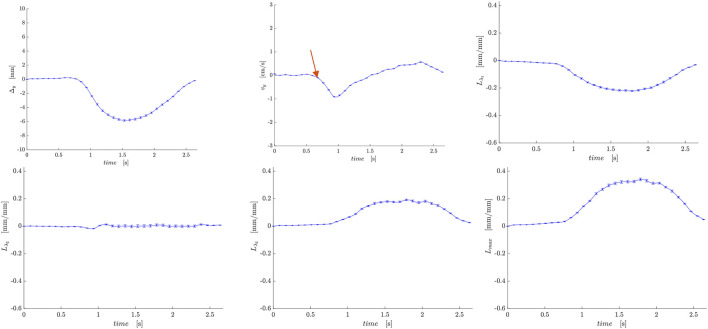
Temporal plots of muscle kinematics extracted from a ROI placed in the VM muscle (quadriceps group) in one subject during isometric contraction at 30%MVC. top row (L to R): y- displacement, velocity in the y-direction, *L*_*λ1*_; second row (L to R): *L*_*λ2*,_ *L*_*λ3*_, and *L*_*max*_. The red arrow points to the peak in the velocity in the y-direction during the contraction phase; the velocity is negative in the VM while it is positive in the LBF muscle (Figure 6).

**FIGURE 6:**
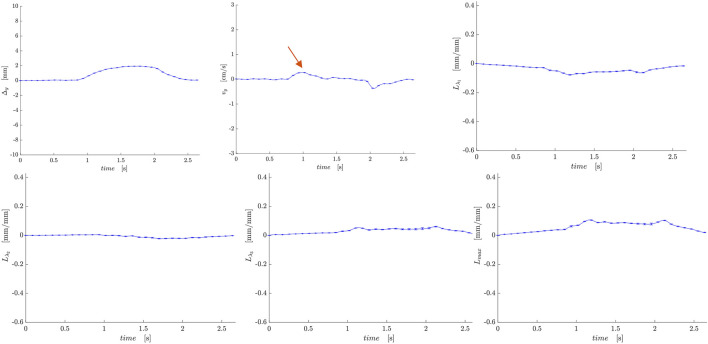
Temporal plots of muscle kinematics extracted from a ROI placed in the LBF muscle (hamstring group) in one subject during isometric contraction at 30%MVC. top row (L to R): y- displacement, velocity in the y-direction, *L*_*λ1*_; second row (L to R): *L*_*λ2*,_ *L*_*λ3*_, and *L*_*max*_. The red arrow points to the peak in the velocity in the y-direction during the contraction phase; the velocity is positive in the LBF while it is negative in the VM muscle (Figure 5).

**FIGURE 7:**
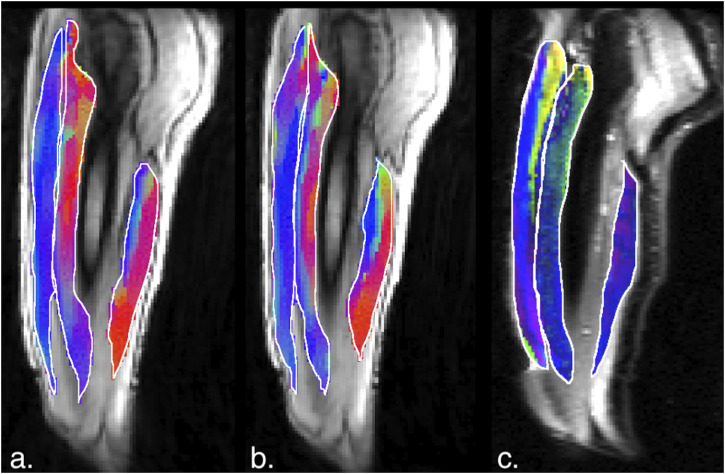
**(a)** Magnitude image with colormaps shown within manually outlined contours of the vastus lateralis, vastus intermedius and the biceps femoris long head muscles (from left to right). The colormap shows the eigenvector direction corresponding to the negative eigenvalue at the temporal frame at the peak of contraction at 45% MVC. The following color mapping scheme is used: red hue (x-component, Medial-Lateral), green hue (y-component, Anterior-Posterior), blue hue (z-component, Superior-Inferior). **(b)** Image similar to that show in **(a)** for the same anatomical location at 30%MVC effort. **(c)** Colormap of the lead eigenvector of the diffusion tensor imaging (DTI) at the location of the dynamic image with the same three muscles manually outlined. This colormap shows the direction of the muscle fibers.

**FIGURE 8:**
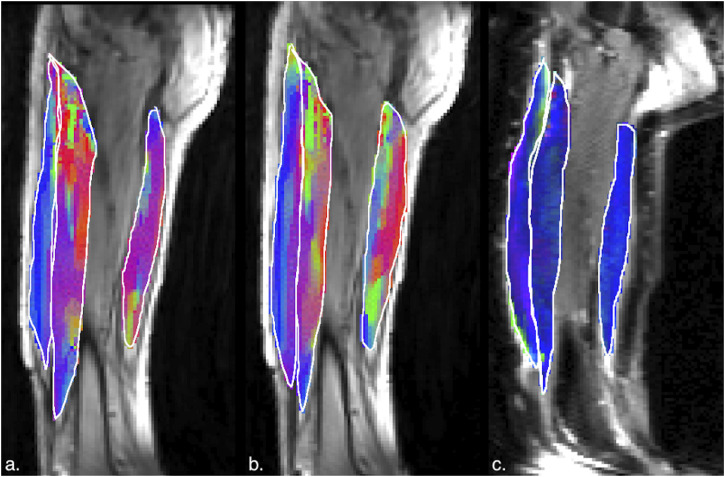
**(a)** Magnitude image with colormaps shown within manually outlined contours of the rectus femoris, vastus intermedius and the semitendinosus and semimembranosus muscles (from left to right). The colormap shows the eigenvector direction corresponding to the negative eigenvalue at the temporal frame at the peak of contraction at 45% MVC. The following color mapping scheme is used: red hue (x-component, Medial-Lateral), green hue (y-component, Anterior-Posterior), blue hue (z-component, Superior-Inferior). **(b)** Image similar to that show in **(a)** for the same anatomical location at 30%MVC effort. **(c)** Colormap of the lead eigenvector of the diffusion tensor imaging (DTI) at the location of the dynamic image with the same three muscles manually outlined. This colormap shows the direction of the muscle fibers.

Figures 5, 6 are temporal plots of muscle kinematics extracted from ROIs placed in the VM muscle and LBF muscles respectively; plots are shown for one subject during isometric contraction at 30%MVC. Of note is that the velocity in the VM (quadriceps) and LBF (hamstring) are opposite in sign indicating the agonist-antagonist activation pattern. Figure 7a is the colormap derived from the eigenvector corresponding to the negative eigenvalue and is shown for one slice at the peak of the contraction cycle at 45% MVC; the colormaps are shown inside the contours of three muscles manually outlined in the magnitude image: from left to right in each image, the contours are VL, VI and LBF. Figure 7b is the colormap from the same slice and temporal frame (as Figure 7a) at 30% MVC. The colormap of the lead eigenvector of the diffusion tensor imaging (DTI) in a matching slice is shown in Figure 7c. It should be noted that the DTI suffers from susceptibility induced artifacts arising from the fact that these images were acquired with a large FOV in the sagittal plane to cover the length of the thigh. The muscles were manually contoured independently on the baseline image of the DTI and the colormaps are shown inside the contours of VL, VI and LBF muscles. The DTI colormap confirms that the fibers in the thigh are aligned predominantly in the superior-inferior direction. Figures 8a–c shows images arranged similarly to Figures 7a–c for the same subject but at a different anatomical location. The manual contours of the muscles in Figure 8 refer to the following from left to right: RF, VI and ST/SM muscles. Note the quadriceps muscles are labeled as ST/SM as the contoured muscle is possibly a mixture of both.

Tables 1,2 list the strain indices for the muscles in the quadriceps and the hamstrings respectively. Table 3 lists the strain indices for the average of all muscles within the quadriceps and the hamstrings. Significant differences in *L*_*λ1*_, *L*_*λ3*_, *L*_*max*_ and *L*_*vol*_ of the quadriceps muscles were found with %MVC (higher strain values at the higher %MVC). Significant differences in *L*_*λ3*_ and *L*_*max*_ of the hamstring muscles were found with %MVC (higher strain values at the higher %MVC). There were no significant inter-muscle differences in the hamstring or quadriceps muscles. The comparison of the averaged values of the quadriceps and hamstring muscles showed significant differences between the two muscle groups for all strain indices (average quadriceps values higher than average hamstring values) and significant differences with %MVC in all the strain indices. It is acknowledged that the variance in the strain measurements is high which affords poor statistical power for detecting small regional differences in the strain indices within the quadriceps and hamstrings muscle groups. However, adequate statistical power to detect significant differences was realized when the four muscles in each muscle group were averaged and the effect size was larger (50% difference between quadriceps and hamstrings). The power calculated using repeated-measures ANOVA logic with a 2 (quadriceps)×2 (force levels) within-subjects design yielded an estimated power of 84% and 55% for detecting differences between the muscle groups and between force levels respectively. This shows that the current study is well powered to detect differences between muscle groups while the power to detect differences in force levels would be improved if the cohort size was increased. Additionally, an increase in the cohort size would also have increased power to detect the smaller regional differences within the quadriceps and hamstring muscles.

**TABLE 1:** Average (over all 12 subjects) strain values in the quadriceps muscles at 30% and 45% MVC.

StrainIndices↓	Vastus medialis		Vastus lateralis		Vastus intermedius		Rectus femoris	
	30 %MVC	45 %MVC	30 %MVC	45 %MVC	30 %MVC	45 %MVC	30 %MVC	45 %MVC
L_λ1_^*^	0.210 ± 0.084	0.217 ± 0.098	0.177 ± 0.043	0.239 ± 0.083	0.185 ± 0.063	0.227 ± 0.075	0.225 ± 0.077	0.272 ± 0.095
L_λ2_	0.029 ± 0.010	0.038 ± 0.028	0.000 ± 0.064	0.010 ± 0.069	0.004 ± 0.075	0.012 ± 0.112	0.022 ± 0.042	0.016 ± 0.066
L_λ3_^*^	0.195 ± 0.063	0.223 ± 0.052	0.200 ± 0.110	0.264 ± 0.181	0.190 ± 0.059	0.225 ± 0.090	0.230 ± 0.089	0.299 ± 0.119
L_max_^*^	0.342 ± 0.113	0.349 ± 0.115	0.331 ± 0.140	0.394 ± 0.227	0.303 ± 0.140	0.393 ± 0.171	0.373 ± 0.179	0.495 ± 0.183
L_vol_^*^	0.001 ± 0.086	0.067 ± 0.147	0.019 ± 0.168	0.073 ± 0.192	0.034 ± 0.111	0.039 ± 0.182	0.062 ± 0.037	0.039 ± 0.125

Mean ± SD, reported for L_λ1_ and median ± IQR, for all other strain variables.

*significant difference between 30% and 45% MVC., note, L_λ2_ was not statistically analyzed as the values were close to noise levels.

**TABLE 2:** Average (over all 12 subjects) strain values in the hamstring muscles at 30% and 45%MVC.

StrainIndices↓	Short head, biceps femoris		Long head, biceps femoris		Semimembranous		Semitendinosus	
	30 %MVC	45 %MVC	30 %MVC	45 %MVC	30 %MVC	45 %MVC	30 %MVC	45 %MVC
L_λ1_	0.132 ± 0.086	0.109 ± 0.160	0.076 ± 0.043	0.103 ± 0.160	0.088 ± 0.066	0.150 ± 0.098	0.079 ± 0.053	0.102 ± 0.057
L_λ2_	0.010 ± 0.034	0.017 ± 0.044	0.008 ± 0.025	0.009 ± 0.044	0.002 ± 0.042	0.024 ± 0.017	0.012 ± 0.016	0.017 ± 0.037
L_λ3_^*^	0.120 ± 0.107	0.160 ± 0.306	0.102 ± 0.064	0.108 ± 0.306	0.156 ± 0.122	0.139 ± 0.132	0.076 ± 0.048	0.123 ± 0.075
L_max_^*^	0.186 ± 0.140	0.211 ± 0.375	0.135 ± 0.064	0.182 ± 0.099	0.192 ± 0.184	0.238 ± 0.168	0.129 ± 0.073	0.166 ± 0.094
L_vol_	0.031 ± 0.119	0.095 ± 0.207	0.019 ± 0.094	0.036 ± 0.118	0.029 ± 0.129	0.075 ± 0.092	0.025 ± 0.088	0.069 ± 0.070

Median ± IQR, reported for all other strain variables.

*significant difference between 30% and 45% MVC, note, L_λ2_ was not statistically analyzed as the values were close to noise levels.

**TABLE 3:** Average (over all 12 subjects and all muscles in a group) strain values in quadriceps and hamstring muscles.

StrainIndices↓	Quadriceps average		Hamstrings average	
	30 %MVC	45 %MVC	30 %MVC	45 %MVC
L_λ1_^a,^^	0.200 ± 0.053	0.235 ± 0.061	0.102 ± 0.045	0.127 ± 0.053
L_λ2_	0.001 ± 0.022	0.005 ± 0.028	0.007 ± 0.015	0.015 ± 0.012
L_λ3_^a,^^	0.204 ± 0.052	0.256 ± 0.067	0.122 ± 0.055	0.163 ± 0.072
L_max_^a,^^	0.333 ± 0.087	0.405 ± 0.103	0.180 ± 0.079	0.230 ± 0.103
L_vol_^a,^^	0.011 ± 0.028	0.018 ± 0.044	0.025 ± 0.061	0.064 ± 0.052

Mean ± SD, is reported for all values.

^a^significant difference between 30% and 45% MVC.

^significant difference between hamstring average and quadriceps average. Note, L_λ2_ was not statistically analyzed as the values were close to noise levels.

## 4 Discussion

This is the first report of volumetric dynamic imaging of thigh muscle under isometric contraction. Volume acquisition was enabled by the compressed sensing accelerated 4D Flow sequence with undersampling by a factor of 7.7. An earlier study reported a randomly under-sampled flow sequence with compressed sensing reconstruction (Mazzoli et al., 2018) to map strain rate in calf muscles during active dorsiflexion/plantarflexion task. Jensen *et al*. used sequential acquisition of 2D slices with 2D VE-PC to compute the volumetric strain tensor in the tibialis anterior under passive plantarflexion of calf muscles (Jensen et al., 2015; Jensen et al., 2016). Malis et al. also used sequential acquisition of 2D slices to extract 3D strain tensors in the MG and soleus muscles under isometric contraction and applied it to studying changes with age (Malis et al., 2020b). The drawbacks of sequential acquisitions are the potential for gross motion between successive acquisitions. Karakuzu et al., performed 3D volume imaging in the ‘undeformed’ (relaxed) and ‘deformed’ state (sustained plantarflexion at 15% MVC) to generate strain tensors in the MG (Karakazu et al., 2017). The strain tensors were then rotated along the direction of muscle fiber tracts to obtain strains along muscle fascicles. While this is an interesting approach to mapping strains, the method is not a dynamic acquisition and it would be difficult to obtain sustained contractions at greater than 15% MVC.

Care was taken to exclude/reposition subjects with visibly gross motion but there is a potential for small gross motion especially at higher %MVC

These translate to small displacements of the entire muscle and thus will not affect the spatial gradient of displacements (all points move together in displacement due to motion as opposed to a deformation of the muscle fiber) required to compute the strain. However, if the motion is large, this manifests as motion artifacts along the phase encode direction and the image has multiple ghosts precluding analysis. Velocity computed from the phase images will be influenced by noise and this will also reflect in the displacement images derived from the velocity images. An analysis performed in an earlier study showed that, by the propagation of errors (Sinha et al., 2018), the noise in the original phase (velocity) images is increased by a factor of 8 in the strain images; thus, denoising of the velocity images is critical to reduction of noise in the strain maps. In the current paper, noise in the phase images was reduced by 3D anisotropic diffusion filtering. Further, compared to earlier studies with single slice and imaging at 1.5 T (Malis et al., 2018), the current study also leverages the increase in signal from volumetric acquisition and 3T imaging. Denoising methods in other studies include a 3x3 median filter to denoise single slice velocity images prior to extracting strain (Delgianni et al., 2017). Image processing strategies to reduce noise in the strain computation have also been proposed. One promising approach is the Local Maximum-Entropy (LME) approximation scheme which computes the spatial gradient of the displacement at a given voxel as the weighted average over 20 voxels around the central voxel; this provides a high degree of denoising in the strain images (Verzhbinsky et al., 2020). However, it should be noted that in addition to technical sources of noise, the variance in the strain indices has an important contribution from physiological differences between subjects. This has also been noted in other muscle function studies (Karakazu et al., 2017).

A 3D ultrasound study reported the fascicle strain in the VI muscle using a cross-correlation based displacement estimation algorithm (Gijsbertse et al., 2017). The latter study was conducted at 30% MVC and determined that deformation in the three orthogonal directions was different for isometric contraction; with −7% along the contraction direction, 20% and-12% in the fiber cross-section. In the current study, the VI has an average compressive strain of ∼19% at 30%MVC which is much larger than that reported in the ultrasound study (Gijsbertse et al., 2017). One potential reason for the discrepancy may arise from the frame of reference for the strains: the current study reports the values in the principal reference frame (eigenvalues reflect the maximum strain values) while the ultrasound study used the fascicle frame of reference. Prior studies have shown that the principal reference frame is not aligned with the fascicles (Sinha et al., 2015; Englund et al., 2011) and thus, only a component of the compressive strain may have been measured in the ultrasound study. However, methodological differences between the two modalities as well as in subject positioning may also contribute to the different values of strains. Deligianni et al., used 2D VE-PC imaging with electrical stimulation at different current amplitudes and reported strains in the vastus lateralis around 11% for a current level estimated to be equivalent to ∼56% MVC (Deligianni et al., 2017). The same group reported strain values of ∼13.8% in a young cohort in the VL and VI muscles under electrical stimulation (Deligianni et al., 2020). These latter strain values are somewhat lower than that seen in the voluntary contractions reported in this study at a lower MVC (45%) but these differences may arise from the differences in the paradigms (stimulation vs voluntary). Other muscle dynamic MRI studies have focused on the medial gastrocnemius, soleus, or anterior tibialis. Principal negative strains in the deep compartment were reported at ∼ −40% and ∼ −24% in the superficial compartment of the anterior tibialis during 50% MVC isometric dorsiflexion contractions (Englund et al., 2011). An earlier study based on sequential 2D VE-PC acquisition reported ∼33% strain in the soleus and ∼37% strain in the MG for 40% MVC isometric contraction. The values in the calf muscles are somewhat higher than strains in the quadriceps muscles at 45% MVC in the current study. However, these differences may be due to the different muscles evaluated (calf vs. thigh) as well as the different contraction paradigms.

The second eigenvalue, which represents the strain in the fiber cross-section perpendicular to the plane of the muscle fibers, is close to zero in the quadriceps and hamstring muscles. Asymmetry of transverse deformation during a dynamic contraction has been reported on calf muscles from 3D MRI studies: anterior tibialis during isometric dorsiflexion (Englund et al., 2011) and in the medial gastrocnemius and soleus during isometric plantarflexion (Malis et al., 2020a; b). Further, dual-probe ultrasound imaging identified transverse anisotropy in the lateral gastrocnemius during cyclic plantarflexion contractions (Randhawa and Wakeling, 2018). Englund et al., hypothesized that the asymmetry in transverse deformation arose from architectural heterogeneity (Englund et al., 2011). Hodgson et al., used computational modeling to predict that the force output of the muscle is higher with increasing anisotropy of transverse deformation and suggested that incorporation of tensile materials oriented along the through-plane axis of the fiber may limit relaxation in that direction (Hodgson et al., 2012). Randhawa et al., suggested that the transverse deformation of the muscle fascicles depends on the stiffness of the aponeuroses, properties of connective tissue structures surrounding muscle, and compressive forces both internal and external to the muscle (Randhawa and Wakeling, 2018). While asymmetry of deformation appears to be a feature of all the lower extremity muscles, the consequences of this on muscle function remain largely unexplored.

Strain indices increased in magnitude with %MVC in both the quadriceps and hamstring muscles and reached significance for some of the strain indices. An increase of the strain indices with %MVC is anticipated; a larger contraction (larger negative strain) will result in a higher force. While it is difficult to comment on linearity of strain with %MVC with two data points, the change going from 0% to 30% MVC is higher than going from 30%MVC to 45%MVC. This decrease with % effort at higher MVCs was also seen in calf muscles (Malis et al., 2020a; b). Ultrasound studies showed that architectural parameters change rapidly to 30% MVC, but change little at higher levels of contraction (Hodges et al., 2003; Li et al., 2014) while simultaneous EMG measurements revealed a linear change with %MVC reflecting the changes in muscle activity (Hodges et al., 2003). Further, a 2D biplane US speckle tracking study reported strain imaging of the *biceps brachii* at 30, 60, 100 %MVC (Lopata et al., 2010) and identified an approximately linear correlation between force and strain. However, the latter study also revealed that the initial slope between 0% and 30% was steep with smaller increases seen for the higher %MVCs. The current findings in thigh muscles with %MVC are thus similar to earlier findings on calf muscle and on the *biceps brachii* in that there is a larger increase of strain from 0% to 30% MVC with smaller increments beyond that. Significant inter-muscle strain differences were not seen in either the quadriceps or hamstring muscles as anticipated based on differences in muscle fiber type composition. However, even though the strain was not significantly different between the different muscles of the quadriceps (or hamstrings), the force produced by each muscle will be different. Future studies can extend the current strain measurements to computation of individual muscle force by including architectural information on physiological cross-sectional area (PCSA) and pennation angle of the muscle.

Coactivation is most commonly detected using multichannel surface electromyography (sEMG). sEMG measures a muscle’s response (in the form of electrical activity) to incoming stimuli (action potentials) from motor neurons, and represents an average of neural drives to multiple motor units (sEMG) (Csapo et al., 2015). In contrast, the 4D Flow MRI evaluated in this paper enables the quantitation of strain associated with a contraction in muscle tissue. While both sEMG and 4D Flow measure muscular activity, there are differences in the muscle activity estimated from these two techniques. sEMG measures the electrical potential generated by neurologically activated muscle fibers, while 4D Flow MRI measures velocity from which displacements and strains can be computed. Strain will be affected not only by neural activation but by other muscle parameters as well, including fiber type composition (Larsson and Moss, 1993); muscle architecture (Azizi and Deslauriers, 2014); and the material properties of the extracellular matrix of the muscle (Hodgson et al., 2012), which allows for the lateral transmission of force and displacement.

The agonist/antagonist behavior can be seen in the temporal plots of the velocity where the velocity is positive in the quadriceps muscles in the contraction phase while it is negative in the hamstrings; the latter finding indicates that when the quadriceps are contracting, the hamstrings are lengthening (coactivation). It should be noted that the incompressibility of muscle tissue combined with transverse asymmetry of muscle deformation leads to a negative eigenvalue and a positive eigenvalue with nearly equal magnitudes at each voxel. When the muscle is contracting during the dynamic cycle, the direction of the negative eigenvalue (contraction) will be approximately along the fiber direction and the direction of the positive eigenvalue will be perpendicular to the fiber, i.e., in the fiber cross-section. The opposing action of the hamstrings to that of the quadriceps is seen in the colormaps of the eigenvectors corresponding to the compressive (negative) strain in the contraction phase of the dynamic cycle. The colormap of the negative strain eigenvector at contraction shows the VL and RF (quadriceps) muscles have a predominantly blue hue indicating the strain eigenvector is along the superior- inferior direction while the hamstring muscle (LBF and ST/SM) have a predominantly red hue (albeit with some heterogeneity) indicating the strain eigenvector is along the medial-lateral direction. This indicates that positive strain exists along the muscle fibers of the hamstrings (lengthening) during the contraction cycle when the quadriceps muscles are contracting. The VI muscle has a more heterogenous colormap at both anatomical locations. However, as visualized in the diffusion colormap, the VI fiber orientation has a blue to blue-green hue as one traverses from distal to proximal. This, combined with the fact that fibers are known to rotate on isometric contraction could potentially cause the strain colormaps to reflect the rotated fiber direction at the peak and not at the start of the contraction (time point at which the DTI was acquired). The extent of fiber rotation is also likely to be dependent on the muscle. Future work will be focused on reorientating the DTI derived eigenvectors using the displacements derived from the dynamic imaging to compute the fiber orientation at the peak of the contraction. It is acknowledged that the strain colormaps are noisy; this arises from the fact that noise is amplified when spatial gradients of displacements are computed to extract strain. Anisotropic diffusion filtering of the phase images as well as the colormaps served to reduce some of the noise; future work will be focused on further reducing noise. However, the fact that the colormap of the strain eigenvector is similar for 30% and 45% MVC indicates that these are consistent and robust determination of the orientation.

Significant differences were found between the quadriceps and hamstrings in all the strain indices when comparing the strain indices averaged over all muscles of the quadriceps to the corresponding averages of the hamstrings. The strain values in the hamstring muscles were lower by a factor of ∼0.5. This level of coactivation is higher than that found from EMG studies of antagonist hamstring activation during concentric quadriceps contraction at 15% of the agonist hamstring activation at eccentric hamstring contraction at MVC (Alkjær et al., 2012). Some of the differences may arise from the different modalities as well as the contraction paradigms (isometric vs concentric) but the main reason may arise from the definition of the coactivation index in the EMG studies as opposed to the simple ratio of strains in the antagonist/agonist muscles reported in the current study. It is also possible that some of the measured strain in the agonist hamstring muscles may arise from intermuscular myofascicular force transmission (Rijkelijkhuizen et al., 2007). This latter type of transmission has been experimentally observed between not only in synergestic muscles but also between agonist/antagonist pairs (Rijkelijkhuizen et al., 2007). However, it should be emphasized that the strain in the antagonist muscle is not arising from a gross mechanical displacement; the latter effect will be seen as a displacement and not as a strain in the muscle. The functional implications of the negative and positive strain eigenvalue are that the former is proportional to the force generation ability of the muscle while the latter potentially reflects the properties of connective tissue structures surrounding muscle. The differences in the negative strain indices between the agonist and antagonist muscles have functional implications for joint stabilization (and injury prevention) as detailed below.

Coactivation of the hamstrings during quadriceps contraction is necessary for joint stability, even in normal individuals—serving to dynamically counteract the anterior pull of the quadriceps on the tibia through assisting the passive stabilizer, the ACL (Yanagawa et al., 2002; Wu et al., 2017). Prior studies indicate that during voluntary contractions of the quadriceps muscle (knee extensors) the hamstrings (antagonist muscles) provide a mechanical contraction opposing the agonist muscles modulated by variations in joint angular displacement providing a mechanism for maintaining joint stability (Solomonow et al., 1987; Wu et al., 2017). Aagaard et al proposed that the antagonist hamstring moments potentially counteract the anterior tibial shear and excessive internal tibial rotation resulting from the contractile forces of the quadriceps near full knee extension. They propose that hamstring coactivation likely supports the mechanical and neurosensory functions of the anterior cruciate ligament (ACL) (Aagaard et al., 2000). In ACL-deficient subjects, an increase in coactivation was observed which is postulated to be a compensatory strategy to provide stability to the knee joint in the anterior-posterior plane during isolated knee extension (Alkjaer et al., 2012). Further, in subjects with osteoarthritis (OA), antagonist coactivation increases and a longitudinal relationship was identified between antagonist hamstring coactivation during isokinetic knee extensor testing and worsening of cartilage morphology over 24 months in men with or at risk for knee OA (Murphy et al., 2022). Earlier studies also revealed that while physiological levels of coactivation serve to stabilize, greater hamstring coactivation is associated with lower knee extensor strength and higher levels of coactivation during walking lead to increased joint loading. The studies discussed above used sEMG to monitor muscle activation; sEMG is often difficult to detect and interpret correctly and can only measure activation in muscles close to the surface of the thigh and may be corrupted by cross-talk from distant muscles. Strain is a measure of mechanical activity and is more directly related to force production than electrical activity from EMG. Further, volumetric MRI can measure strain in all the muscles. Future studies using the currently reported technique to determine coactivation could potentially help in characterizing and monitoring disease conditions such as ACL deficiency or OA. The findings on normal subjects can be translated to assessing subjects with ACL-deficiency or osteoarthritis conditions since the contractions are performed at submaximal force levels. Further technical improvements in accelerating image acquisition for example, by using deep learning-based reconstruction techniques (Vishnevsky et al., 2020) would increase the potential to extend the current technique to clinical populations.

Shear strain indices increased significantly with %MVC in both the quadriceps and hamstrings in the current study and was the highest absolute value among all the strain indices. Prior 2D strain rate studies identified shear strain rate to be significantly different between age groups and further, emerged as a significant predictor of force loss with age or with disuse atrophy (Malis et al., 2018; Sinha et al., 2018). The physiological origin or significance of the shear strain experimentally measured from the MRI studies is still unknown. However, some inferences can be made from computational modeling studies that proposed shearing in the endomysium as the mechanism of lateral transmission of force (Sharafi and Blemker, 2011); the experimentally measured shear strain from MR may be a measure of the shear in the endomysium and thus a surrogate marker of lateral transmission of force. Future studies will help identify the clinical utility of thigh muscle shear strain in subjects with musculoskeletal diseases (Schlaffke et al., 2024). The physiological implications of the significant differences in shear strain as well as in the positive eigenvalue, *L*_*λ3*_, between agonist and antagonist muscles may reflect differences in the material properties of the extracellular matrix and is hypothesized to affect the lateral transmission of force.

The other invariant computed from the strain tensors is the volumetric strain which is the fractional change in volume. In a nearly incompressible tissue like muscle, this index should be close to zero. Indeed, compared to strain eigenvalues, the volumetric strain is an order of magnitude smaller. Interest in the volumetric strain stems from the fact that it is inversely related to intramuscular pressure; the latter measure has been shown to be linear with force and potentially a marker of individual muscle force (Reinhardt et al., 2016). Volumetric strain in the individual muscles did not show any significant change with %MVC; however, the averaged values over the quadriceps and the hamstrings showed a significant change with %MVC as well between the hamstring and the quadriceps muscle groups. The volumetric strain in the hamstrings and quadriceps at both % MVCs were positive, increased with % MVC and was higher in the hamstrings. Earlier studies extracted volumetric strain from the anterior tibialis under passive plantarflexion ((Jensen et al., 2015; Jensen et al., 2016); the latter studies revealed predominantly positive volumetric strains with a heterogeneous distribution under passive stretch. Jensen et al. reported median values of 0.056 for the volumetric strain in the anterior tibialis (Jensen et al., 2016), which is in higher than seen in the current study. A surprising finding is that volumetric strain is positive and increases with %MVC. However, *L*_*vol*_ should be interpreted with caution since it is the sum of two quantities with opposite signs but whose absolute values are very close to each other (*L*_*λ1*_, *L*_*λ3*_). Future studies are needed to identify if *L*_*vol*_ is a reliable measure and a potential surrogate marker of intramuscular pressure as well to discern the functional implications of the significant differences in *L*_*vol*_ between the quadriceps and hamstring muscles.

The study has limitations: there were only 12 subjects in the study. While the cohort was fairly well defined, the self-report on their physical activity may have been subjective. This might have caused the larger range in MVC force across subjects. The second limitation is that the peak values were extracted from a VOI placed within each muscle whereas the strain is spatially heterogenous. The VOI approach was chosen so that measurements were made in fairly uniform regions of the strain indices, avoiding areas of steep gradients. Future work will focus on measuring the strain indices in sub-regions of the entire muscle volume to characterize the heterogeneity of the strain patterns. Exploration of the relationship of the strain indices to %MVC requires more than the two %MVCs. However, the prone position for thigh imaging was challenging to complete for even two %MVCs. Higher acceleration and consequent faster scanning may help in acquisitions at three or four %MVCs.

## Data Availability

The raw data supporting the conclusions of this article will be made available by the authors, without undue reservation.

## Supplementary material

SUPPLEMENTARY VIDEO 1
Video of displacement maps of study with and without motion artifacts.

SUPPLEMENTARY VIDEO 2
Video of the principal strains and shear strain at one spatial location.

SUPPLEMENTARY VIDEO 2
Video of the compressive strain at several spatial locations.

SUPPLEMENTARY DATA SHEET 1
Supplementary Figures and Video Legends.
