# Supplementary material for: Strain mapping using compressed sensing accelerated 4D flow MRI—Potential for detecting coactivation in thigh muscles
Source: Front Physiol. 2025 May 23;16:1583024. doi: 10.3389/fphys.2025.1583024 (PMC12142688; doi:10.3389/fphys.2025.1583024)
Supplement: Supplementary file 4 [file DataSheet1.docx]

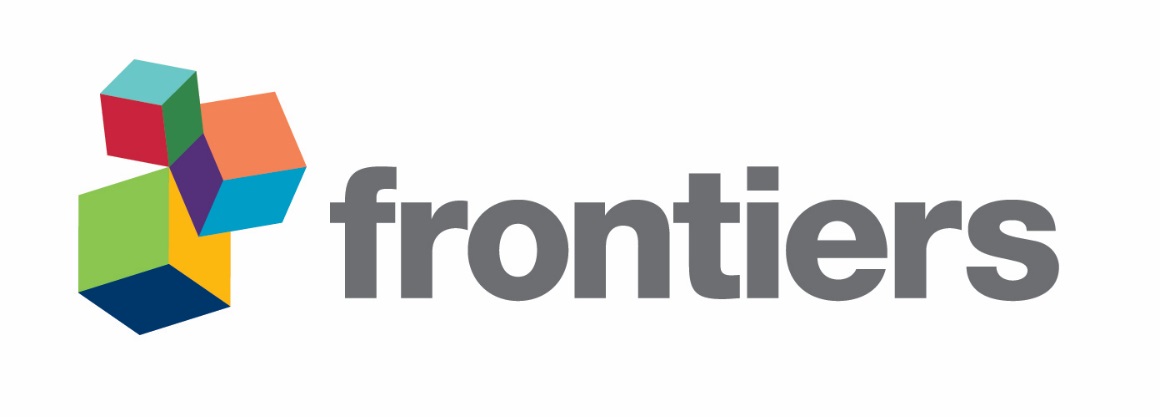


Supplementary Material

# Supplementary Figures

## Supplementary Figures

**Supplementary Figure 1a.** ROI placement in the Quadriceps shown in one of the subjects. The ROIs from (L to R): Rectus Femoris (RF), Vastus Intermedius (VI), Vastus Lateralis (VL), and Vastus Medialis (VM).

**Supplementary Figure 1b.** ROI placement in the Hamstrings shown in one of the subjects. The ROIs from (L to R): Short-Head Bicep Femoris (SBF), Long-Head Bicep Femoris (LBF), Semimembranosus (SM), and Semitendinosus (ST).

**Supplementary Figure 2.** Relative force as a function of the dynamic cycle (period of 3 seconds). The measured force is the output of the force transducer appropriately processed and reflects the force pattern exerted by the subject for each iteration of the acquisition. The left panel shows the plot for a subject with consistent contractions while the right panel is plot from a subject with inconsistent contractions (unable to follow the template curve).


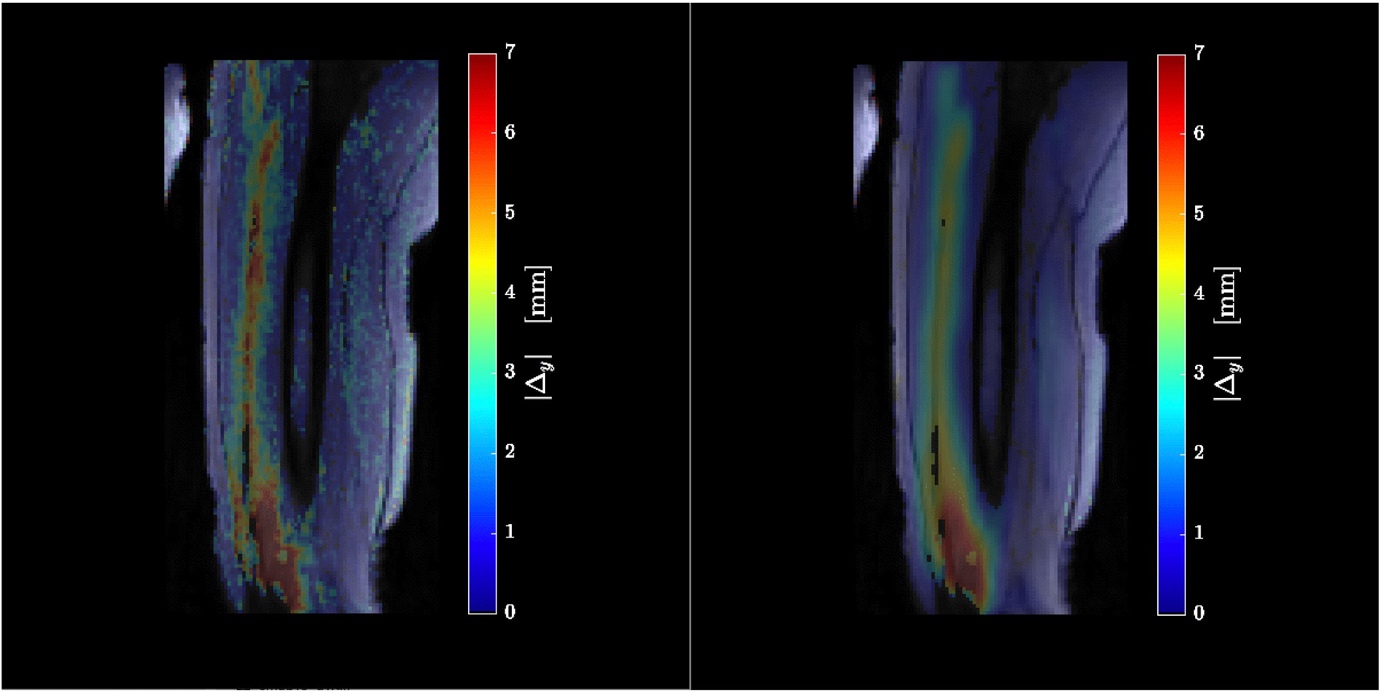


**Supporting Information Figure S3:** Colormap of the Velocity (*v_y_*) shown in one slice at a temporal frame close to the peak of the force curve from acquired velocity data (left image) and after application of the 3D anisotropic diffusion filter (right image). The decrease in noise as well as the preservation of the edges is clearly seen in the denoised images on the right.


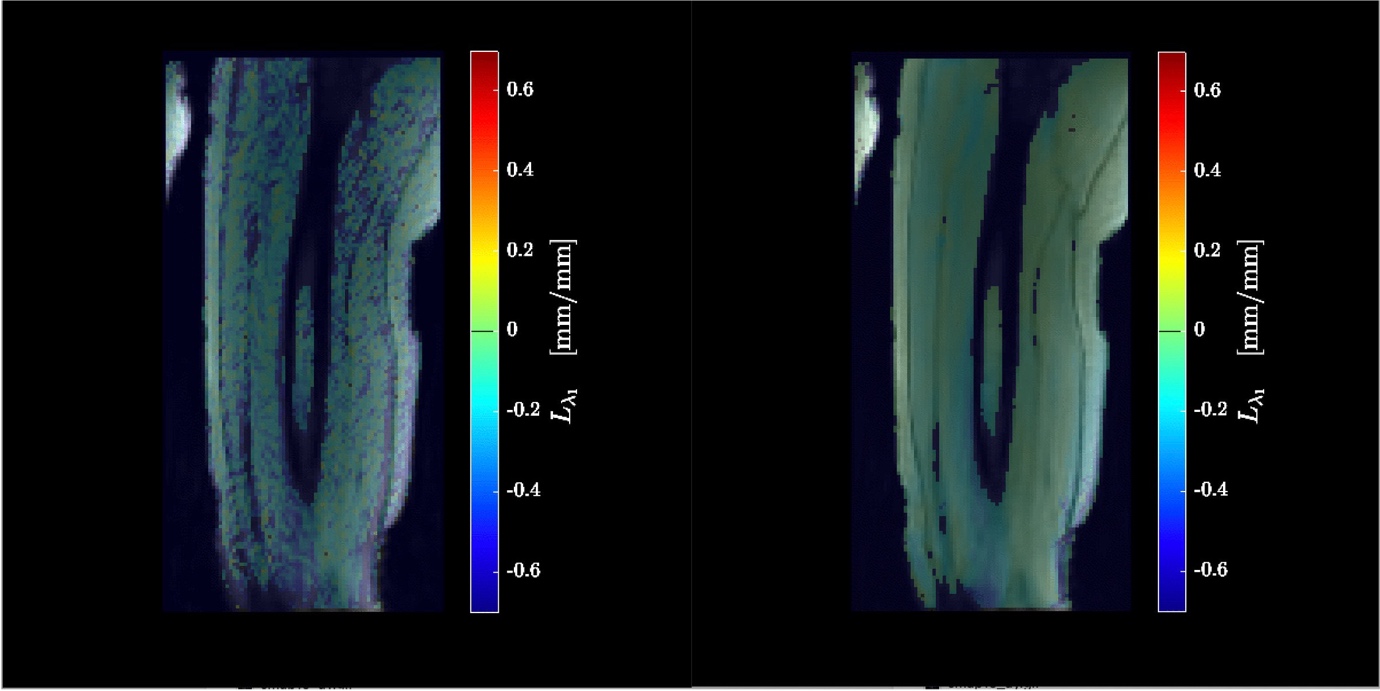


**Supporting Information Figure S4:** Colormap of the compressive strain (*L_λ1_*) shown in one slice at a temporal frame close to the peak of the force curve from strain computed from acquired velocity data (left image) and strain computed after application of the 3D anisotropic diffusion filter (right image). The increase in noise in computed strain images compared to the velocity images is obvious specially in the original noisy velocity images (compare Figs. S3 and S4, left images). As in the case of the velocity images, the decrease in noise as well as the preservation of the edges is clearly seen in the strain computed from the denoised velocity data.

# Supplementary Videos

**Supplementary Video 1:** Shows images for a subject with incorrect (left panel) and effective restraint (right panel) at the knee respectively. Within each panel, the left image is the magnitude image at the temporal frame corresponding to the peak of the force curve and on the right is a video of the x-displacement maps through the dynamic cycle. The gross motion in the incorrectly restrained subject is seen in the motion artifacts in the magnitude image at the peak of the force curve as well as the in the video where large x-displacements can be seen.

**Supplementary Video 2:** Shows the strain maps (*L_λ1,_ L_λ2_, L_λ3,_ L_max_*) for one slice from a subject’s volumetric thigh dynamic acquisition at 45% MVC through the dynamic cycle of 32 temporal frames. The strain values follow the force curve and can be seen to peak (darker red (positive strain) or blue (negative strain) hues. Compressive strains are displayed on the *L_λ1_* maps while lengthening strains are displayed on the *L_λ3_* maps. Video of the maps of the second eigenvalue, *L_λ2_*, representing the deformation orthogonal to *L_λ1_* and *L_λ3_* and in the fiber cross-section display values close to zero strain. Video of the maps of the shear strain, *L_max_* , shows that this index has the largest values.

**Supplementary Video 3:** Shows the compressive strain maps (*L_λ1_*) for representative slices of a subject’s volumetric thigh dynamic acquisition at 45% MVC through the contraction cycle. The spatial pattern of the compressive strain in the different thigh muscles can be seen as the volume is panned.

.
